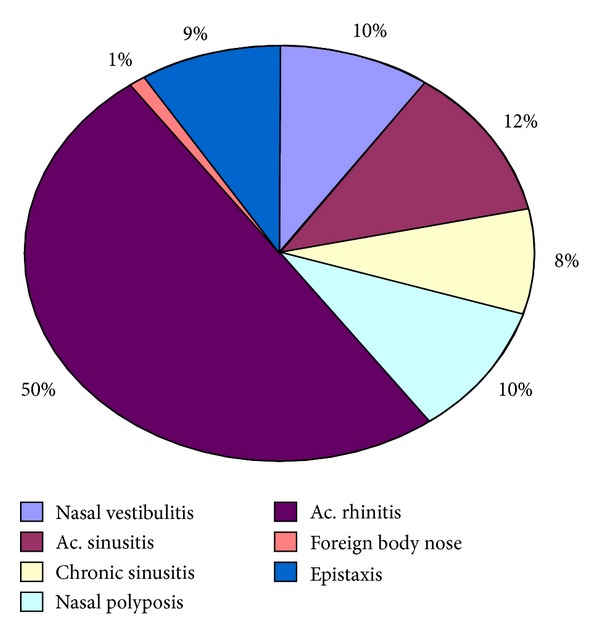# A Model for Provision of ENT Health Care Service at Primary and Secondary Hospital Level in a Developing Country

**DOI:** 10.1155/2013/562643

**Published:** 2013-09-03

**Authors:** Lingamdenne Paul Emerson, Anand Job, Vinod Abraham

**Affiliations:** ^1^Department of ENT, UNIT-I, Christian Medical College, Vellore 632001, India; ^2^Department of Chad, Christian Medical College, Vellore 632001, India

## Abstract

ENT problems are the most common reason for a visit to a doctor in both rural and urban communities. In many developing countries, there is a lack of ENT specialists and overburdened hospital facilities. To date, there is no comprehensive study that has evaluated the spectrum of ENT disorders in a rural community. *Methods*. A prospective study was done for a period of three years to profile the cases presenting to the outpatient clinic in a secondary care hospital and in the camps conducted in tribal areas in Vellore District of Tamil Nadu, India. Trained community volunteers were used to identify ENT conditions and refer patients. *Results*. A total of 2600 patients were evaluated and treated. Otological symptoms were the most commonly reported with allergic rhinitis being the second most commonly reported. Presbycusis was the most common disability reported in the rural community. The other symptoms presented are largely related to hygiene and nutrition. *Conclusion*. Using trained community workers to spread the message of safe ENT practices, rehabilitation of hearing loss through provision of hearing aids, and the evaluation and surgical management by ENT specialist helped the rural community to access the service.

## 1. Introduction

The most common problems warranting a visit to a doctor or a health care provider in developing countries are related to ear nose and throat (ENT) [1, 2]. ENT problems are the most common problems for which there are home remedies to medical treatments which are available, and most individuals manage their problem in the community without seeking help. In addition, due to the lack of specialist professionals in this field, these problems are treated by community practices. The studies done have looked at the prevalence of ENT diseases in children [3, 4] and have shown that the disease burden is due to otitis media and its sequelae which are the most common causes of preventable hearing loss in children in developing countries [5, 6]. The prevalence of traditional practices increased the disease morbidity requiring surgical management. Postal survey using questionnaire methods was used to assess the prevalence of ENT-related disorders in a community. There is no data on the prevalence of otorhinolaryngological diseases in a rural community in India. The present study was undertaken to determine the prevalence of ENT disorders in population presenting to a secondary care hospital with emphasis on primary care in rural communities and tribal area. This is the first study done in a developing country as proper understanding of the magnitude of ENT diseases and the factors associated with their occurrence in the community is important to enable the formulation of health care services aimed at early detection and treatment.

## 2. Methods

A prospective study was done for a period of three years (2009–2012) to profile the cases presenting to the ENT clinic which was conducted once a week in a secondary care hospital, and once a month in tribal areas of Vellore District. The ENT team comprised of ENT specialist, audiologist, and community workers. The population covered was four lakhs, and the tribal population consisted of thirty thousand. These communities are predominantly farming community. 40% live below the poverty line. Literacy of women is 40% and of men is 60%. 25% are under 15 years. Elderly form 6% of the population. The male : female ratio is 1000 : 980. Being farmers, they are dependent on the rainfall for agriculture, and work is seasonal.

The clinic in secondary care hospital was well equipped with an operating microscope and ENT instruments. An initial programme of training community volunteers was conducted in rural and tribal villages. These community volunteers were taught about safe ear care practices, the harmful effects of prevalent traditional practices, and the treatment options available. They were taught to identify the symptoms of ear, nose, and throat diseases, namely, ear pain with/without discharge, recurrent upper respiratory tract infections, throat pain difficulty swallowing, associated with fever in the community and refer them to the camps conducted periodically. Community hearing workers preferably graduates with science subjects were trained to do hearing assessment using a portable audiometer for adults and fit hearing aids (semidigital, trimmer model) in the community. Children with learning disabilities and delay or lack of speech were identified in the community with the help of school teachers and community hearing workers and were referred to tertiary hospital for detailed evaluation to rule out other disabilities and for rehabilitation. Questionnaire method of data collection was used to know the prevalence of ENT-related conditions in the tribal community, and referred patients received treatment in the camps; surgical cases were referred to secondary and tertiary hospital for further management.

All the patients had undergone a complete clinical examination by the ENT specialist, and appropriate investigations were done depending on the merit of the presenting complaint.

Patients were seen by community health physicians in the secondary care hospital and were referred to the ENT clinic which was conducted once a week. Emergency cases which were referred to the tertiary hospital were not taken for analysis.

### 2.1. Statistical Methods

All the data was entered into Microsoft Excel format and SPSS software. The mean number of patients, both adults and children, calculated, and the ratio of prevalence among both the groups was arrived at. Pearson's chi-square test was done to compare the relationship of otitis media in children with various factors leading to chronic condition. The level of significance was set at *P* less than 0.05.

## 3. Results

In our study, a total of 2600 patients both adults and children were seen during the years 2009–2011, which included a total number of 20 camps that were conducted in tribal areas.

**Figure 1:**
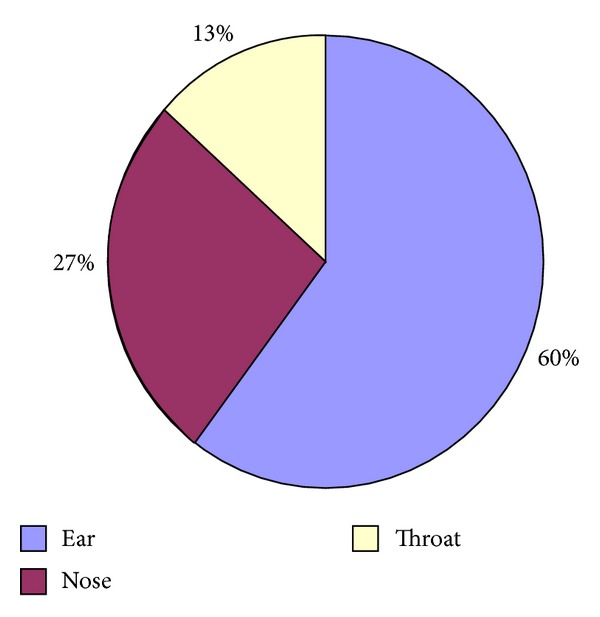


Otological symptoms were the most commonly reported (60%), with pain and ear discharge being reported in pediatric and adult population followed by acute rhinitis due to allergy and infectious causes (Figure 1). Hearing loss was most commonly reported in patients over 50 years of age with moderately severe to severe hearing loss needing rehabilitation.

### 3.1. Ear Disorders

**Figure 2:**
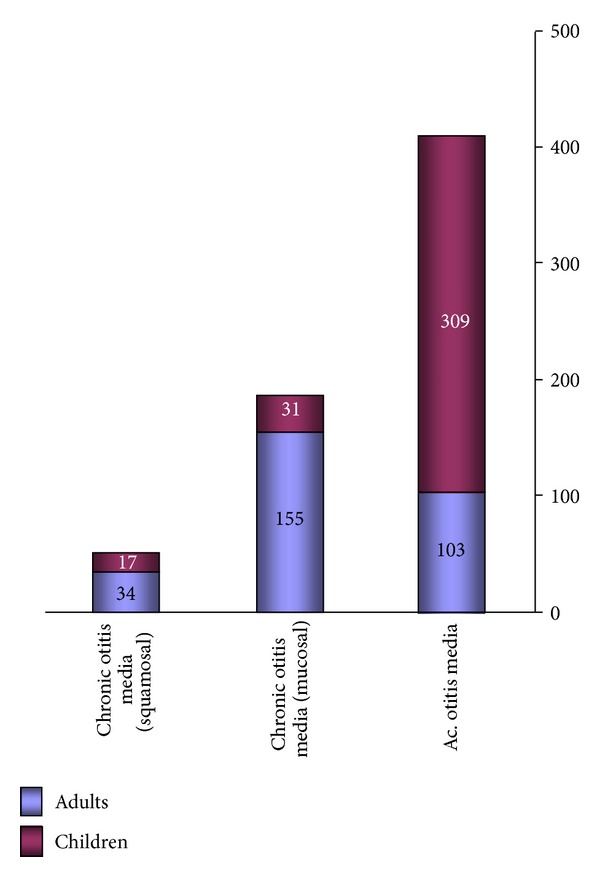


A total of 1526 (*n*) patients were evaluated with various otological problems. Ear pain associated with ear discharge was the most common complaint (49.7%). Acute otitis media was seen in 413 (27%) with children being three times more affected than adults (3 : 1 ratio). Chronic otitis media mucosal disease was seen in 186 (12.1%) with an adult child : ratio of 5 : 1; squamosal disease was seen in 52 patients with adults having a greater prevalence, adult : child ratio of 2 : 1 (Figure 2). 2 patients presented with complications of acute mastoiditis were referred to tertiary hospital. Otitis externa with otomycosis and associated perichondritis was seen in 274 (17.9%) patients, which was mostly due to self-cleaning with sticks and pins. This was equally seen in both adults and children. Presbycusis was the most common complaint among the adult population (19%). Idiopathic Bell's palsy was noted in 11 patients of whom 2 children presented with recurrent episodes of facial paralysis which were referred to tertiary centre, and adults who were affected were treated with steroids and facial physiotherapy. Wax was seen in 10% of the patients.

**Figure 3:**
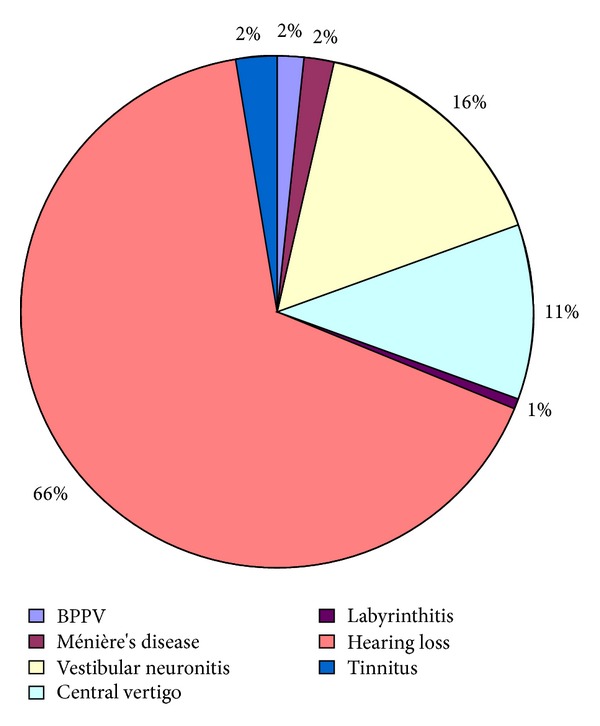


Of the various causes of vertigo evaluated, only 7 (0.4%) had classical BPPV. Followup of patients referred to tertiary hospital for symptoms of vertigo showed vestibular neuronitis in 72, Ménière's disease in 9 and acute labyrinthitis in 3 patients (Figure 3).

### 3.2. Acute Otitis Media

**Figure 4:**
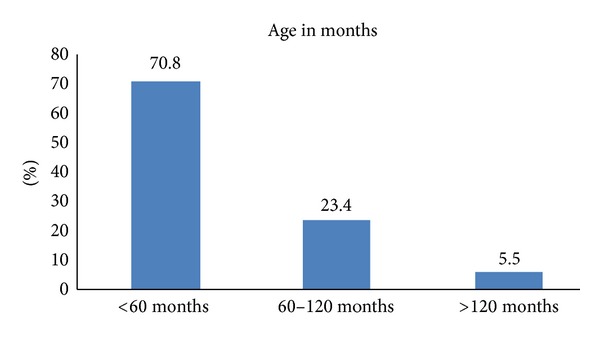


**Figure 5:**
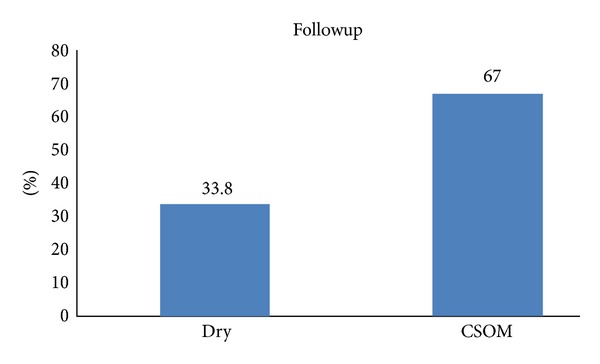


161 children less than 16 years presenting with acute otitis media were followed up. Both boys and girls were equally affected. 33.8% had dry ears, and 67% proceeded to chronic otitis media. Chi-square (0.17) showed that the age of onset is indirectly proportional to the chronicity of the disease; that is, early onset of acute otitis media predisposes to chronic otitis media. However, there was no correlation between episodes of AOM and chronicity of the disease (Figures 4 and 5).

Foreign body in the ear was seen in 10 children.

#### 3.2.1. Treatment

The patients with ear discharge were suction cleaned to identify the pathology whether bacterial/fungal and appropriate treatment was given in the form of antibiotic ear drops/antifungal ear drops. In addition, if there was an associated otitis externa, oral antibiotics to cover staphylococcus and pseudomonas were prescribed. In children, in addition to oral antibiotics, prophylactic antibiotic ear drops were given. Parents were counseled in healthy ear care practices via hand washing, protecting the ear while bathing/swimming. Surgical management of chronic suppurative otitis media was done at secondary care and tertiary hospital.

### 3.3. Nose Disorders

Acute rhinitis was the most common complaint reported by 209 (50%); it has a seasonal predisposition with most of the patients having problems during winter months with equal predisposition among adults and children. 34 (8%) of the patients had recurrent sinusitis which proceeded to features of chronic sinusitis over a period of three years. 2 of the patients were children. Nasal polyposis was seen in 43 (10%) of patients which was surgically treated at tertiary centre. Allergic rhinitis was seen in 35% with peak incidence noted in the months of June, July, and August. This was most commonly seen in adults rather than children. Vestibulitis along epistaxis was seen in 10% of patients mostly in children which was mostly due to nose-picking (Figure 6).

#### 3.3.1. Treatment

Rhinitis of allergic etiology was treated with antihistamines, nasal steroid sprays; patient was counseled on the need of identifying the trigger factors as far as possible to avoid them; however, since most of the patients had symptoms related to their occupation (painting, masonry, and working in dust), medical treatment was needed.

### 3.4. Throat

Various causes of throat pain were evaluated. Recurrent tonsillitis was the most common complaint seen in 140 (49%) of the patients with more than 85% of cases being positive of culture. This was most commonly seen in children with an child : adult ratio of 3 : 1.

Adenotonsillectomy was done almost in all the patients. The peak months were January, February, and June. Pharyngitis comprised the second most common cause 60 (21%) and was most commonly seen in adults. Symptoms of gastroesophageal reflux disease were seen in 26 (9%) of the patients. Aphthous ulcers were seen in 60 (21%) of patients.

### 3.5. Laryngeal Pathology

Among the various patients (*n* = 32) evaluated for change of voice and difficulty swallowing. Vocal cord pathology ranging from acute and chronic laryngitis 14 (19%) to polyps/nodules 12 (16%) was seen in the patients. Difficulty swallowing was evaluated with blood investigations, and barium swallow and 17 (23%) had all the features of plummer vinsons syndrome which was secondary to chronic iron deficiency anaemia which was seen in females (age > 40 years). Other causes of difficulty swallowing were referred to tertiary hospital for further evaluation.

## 4. Discussion

An approach of creating awareness, health education, and treatment availability could be the reason for the large number of disease prevalence reported in this study.

In our study, otological symptoms were the most commonly reported with the major burden being chronic suppurative otitis media as also reported by WHO [7]. There is an indirect relationship between age of onset and chronicity of the disease, and hence risk factors that can be targeted should be identified. Overcrowding and exposure to wood and cigarette smoke should be reduced [8], and poor hygiene should be improved, including access to clean water. Health education messages in relation to personal hygiene could be developed to target known risk factors. Universal immunisation practice should be emphasized in view of increased risk with multiple types of *H. influenzae* and *S. pneumoniae* [9] and a beneficial effect in reducing the number of recurrent attacks [10].

Allergic rhinitis is a disorder which is showing an upward trend in rural communities due to an increase in pollution. Allergic rhinitis is associated with significant comorbidities and health care costs [11, 12] and has been identified as one of the top ten reasons for visits to primary care clinics.

In our study, it was seen that 8% of the patients had progressed to chronic sinusitis; nasal polyposis was also seen in 10% of the patients which was related to chronic allergic rhinitis. This is in agreement with the study done by Ahmadiafshar et al. [13] which showed a correlation between nasal polyposis and duration of symptoms of allergic rhinitis.

It is well known that tonsillitis may be caused by allergens in the food like coloring substance, preservatives, and also cold foods. In our rural community, patients presenting with throat pain, fever, and difficulty swallowing were less; however, the number of patients who progressed to chronic infection is very high. This could be explained on inadequate duration of treatment, consumption of ice creams, and juices with no proper refrigeration.

The cause of oral mucosal ulcers may be related to a temporary weakness in immune system (cold or flu), hormonal changes, mechanical irritation, stress, low levels of vitamin B12, folate, iron, and ferritin [14, 15]. In our study, aphthous ulcers (21%) were treated with multivitamin supplements and saline gargles. Iron deficiency anemia was noted in 23% of patients. This is in agreement with various studies which have reported a high incidence of iron deficiency anemia, vitamin B12, [16], and minerals (micronutrients) [17] in developing countries.

## 5. Conclusion

Creating awareness of the common ENT conditions and how they are caused and treatment options available help decrease the burden of the disease in the community. Using trained community workers to spread the message of safe ENT practices, provides rehabilitation of hearing loss through provision of hearing aids, and the evaluation and surgical management by the specialist (once a week) helped the rural community to access the service.

This model of ENT health care delivery is effective and along with primary care health personal helps in alleviating the disease burden in the rural and tribal communities. This model of providing ENT services could have an impact across the entire nation especially in developing countries where there is lack of awareness and rehabilitation options due to limited resources.

## Figures and Tables

**Figure 6:**